# MicroRNA-495 induces breast cancer cell migration by targeting JAM-A

**DOI:** 10.1007/s13238-014-0088-2

**Published:** 2014-07-30

**Authors:** Minghui Cao, Weiwei Nie, Jing Li, Yujing Zhang, Xin Yan, Xiaoxiang Guan, Xi Chen, Ke Zen, Chen-yu Zhang, Xiaohong Jiang, Dongxia Hou

**Affiliations:** 1 State Key Laboratory of Pharmaceutical Biotechnology, Jiangsu Engineering Research Center for microRNA Biology and Biotechnology, School of Life Sciences, Nanjing University, Nanjing, 210093 China; 2 Department of Medical Oncology, Jinling Hospital, School of Medicine, Southern Medical University, Guangzhou, 510282 China; 3 The Comprehensive Cancer Center of Drum Tower Hospital, Medical School of Nanjing University and Clinical Cancer Institute of Nanjing University, Nanjing, 210008 China

**Keywords:** miR-495, JAM-A, breast cancer, migration

## Abstract

MicroRNAs (miRNAs) are small, non-coding RNAs that function as post-transcriptional regulators of gene expression. The deregulated expression of miRNAs is associated with a variety of diseases, including breast cancer. In the present study, we found that miR-495 was markedly up-regulated in clinical breast cancer samples by quantitative real time-PCR (qRT-PCR). Junctional adhesion molecule A (JAM-A) was predicted to be a potential target of miR-495 by bioinformatics analysis and was subsequently verified by luciferase assay and Western blotting. JAM-A was found to be negatively correlated with the migration of breast cancer cells through loss-of-function and gain-of-function assays, and the inhibition of JAM-A by miR-495 promoted the migration of MCF-7 and MDA-MB-231 cells. Furthermore, overexpression of JAM-A could restore miR-495-induced breast cancer cell migration. Taken together, our findings suggest that miR-495 could facilitate breast cancer progression through the repression of JAM-A, making this miRNA a potential therapeutic target.

**Electronic supplementary material:**

The online version of this article (doi:10.1007/s13238-014-0088-2) contains supplementary material, which is available to authorized users.

## Introduction

Breast cancer is the most common malignancy and the leading cause of cancer death among females worldwide, accounting for ~36% of female primary malignant tumors (Jemal et al., 2011). Although advances in diagnosis such as regular mammography and appropriately systemic treatments have improved the prognosis (DeSantis et al., 2014), distant metastases usually occur several years after the primary breast cancer, causing approximately 90% of breast cancer mortality (Bendre et al., 2003). Considering that the precise mechanisms of breast cancer metastasis remain largely unknown, additional investigation is necessary to clarify the progression of this phenomenon.

MicroRNAs (miRNAs) are a class of endogenous, non-coding RNAs with lengths of 18–25 nt (Bartel, 2004). By base-pairing with 3′-untranslated region (3′-UTR) of messenger RNA (mRNA), miRNAs can repress the expression of target genes by inhibiting translation or by destabilizing the mRNA (Bartel, 2009; Fabian et al., 2010). Under normal circumstances, miRNAs participate in a broad range of biological processes, such as cell proliferation and differentiation, development, metabolism, immunity and stress responses (Ambros, 2001). In recent years, the deregulated expression of miRNAs has been widely reported in many diseases, especially in cancer (Lu et al., 2005; Osada and Takahashi, 2007). With an increasing number of target genes of miRNAs being validated by experimental assays and verified in clinical samples, miRNAs have gradually emerged as a new important regulator of tumorigenesis and have been found to be involved in various aspects of cancer progression including tumor metastasis (Lujambio and Lowe, 2012). For example, miR-10b is specifically up-regulated in metastatic breast cancer cells, resulting in the increased expression of a well-characterized pro-metastatic gene, *RHOC*, by inhibiting the translation of HoxD10 (Ma et al., 2007). Moreover, the expression of miR-206 is selectively reduced during breast cancer metastasis, and this reduction is positively correlated with a low metastasis-free survival in patients (Li et al., 2013; Vimalraj et al., 2013). These findings highlight the need for thorough investigations of miRNAs that are aberrantly expressed during breast cancer progression, especially miRNAs associated with breast cancer metastasis (Vimalraj et al., 2013).

In this study, we demonstrated that miR-495 is significantly up-regulated in primary breast cancer tissue samples when compared with noncancerous tissue samples. By manipulating miR-495 levels in MCF-7 and MDA-MB-231 cells, we proved that miR-495 promotes the mobility of breast cancer cells. JAM-A was predicted to be a target of miR-495, which was verified by luciferase assay and Western blotting; the function of *JAM-A* in breast cancer metastasis was validated by overexpression or knock down of the JAM-A protein. Finally, the rescued expression of JAM-A could reverse the observed effects of miR-495. Our study demonstrates that miR-495 acts as a metastasis promoter by directly targeting JAM-A, suggesting that miR-495 has potential therapeutic value for breast cancer treatment.

## Results

### MiR-495 is up-regulated in clinical breast cancer specimens and is positively correlated with the mobility of breast cancer cells

**Figure 1:**
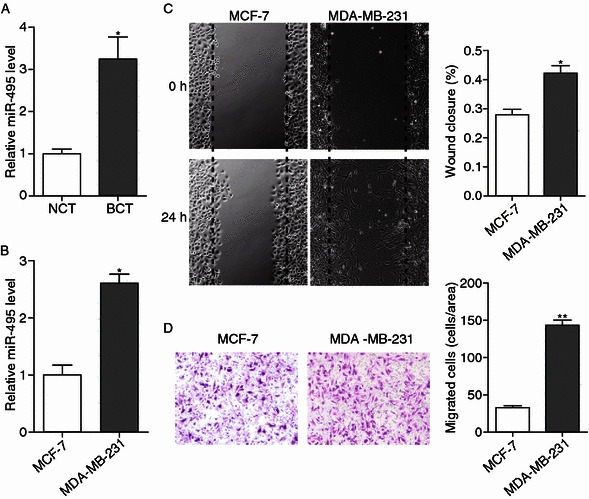
**The expression of miR-495 was increased in breast cancer tissues and was positively correlated with the mobility of breast cancer cells**. (A) Quantitative real time-PCR analysis of the relative expression of miR-495 in seven pairs of breast cancer tissue (BCT) and non-cancerous tissue (NCT) samples. (B) Quantitative real time-PCR analysis of the relative expression of miR-495 in breast cancer cell lines MCF-7 and MDA-MB-231 cells. (C) Left panel: Representative image of wound healing assay of MCF-7 and MDA-MB-231 cells. Right panel: Quantitative analysis of the wound closure rates. (D) Left panel: Representative image of Transwell assay of MCF-7 and MDA-MB-231 cells. Right panel: Quantitative analysis of the migration rates. *P < 0.05; **P < 0.01

First, the level of miR-495 in clinical breast cancer tissue samples was determined using quantitative real time-PCR (qRT-PCR), and we found that the level of miR-495 in breast cancer tissues was markedly higher than in paired adjacent normal breast tissues (Fig. 1A), suggesting that miR-495 is associated with the progression of breast cancer. The level of miR-495 in two different breast cancer cell lines MCF-7 and MDA-MB-231 cells was then detected, and we found that miR-495 was significantly up-regulated in MDA-MB-231 cells (Fig. 1B). MDA-MB-231 cells exhibited a higher mobility in wound healing assays and Transwell assays (Fig. 1C and 1D), indicating that miR-495 was positively correlated with the mobility of breast cancer cells.

### JAM-A is a potential target of miR-495 in breast cancer cells

**Figure 2:**
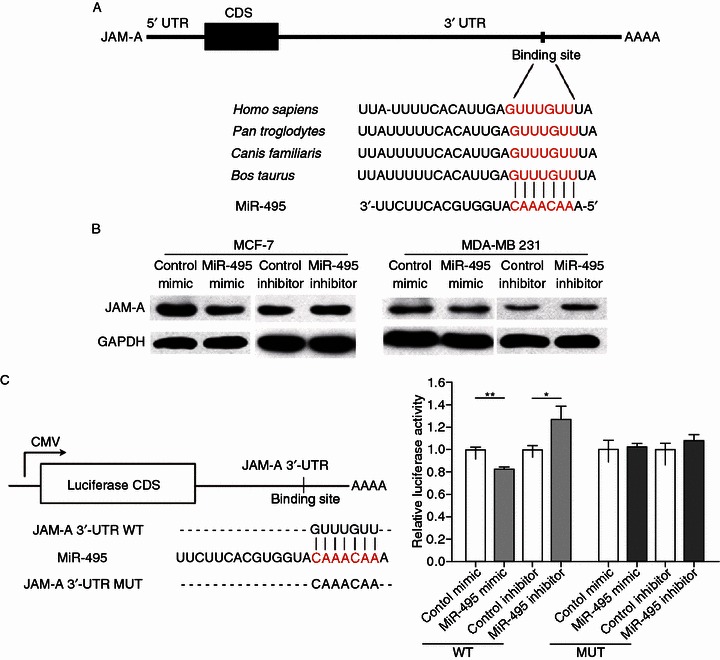
**JAM-A is a target gene of miR-495 in breast cancer cells**. (A) Schematic illustration of the conserved miR-495 binding sites. The JAM-A 3′-UTR contains one predicted miR-495 binding sites. The seed regions of miR-495 and the seed-recognizing sites in the JAM-A 3′-UTR are indicated in red, and all nucleotides in seed-recognizing sites are completely conserved across several species. (B) Western blotting analysis of JAM-A protein levels in MCF-7 and MDA-MB-231 cells transfected with miR-495 mimic or inhibitor. (C) Direct recognition of the JAM-A 3′-UTR by miR-495. Firefly luciferase reporters containing either wild-type (WT) or mutant (MUT) miR-495 binding sites in the JAM-A 3′-UTR were co-transfected into MDA-MB-231 cells with the scrambled negative control RNA, miR-495 mimic or inhibitor. At 24 h post-transfection, the cells were assayed using a luciferase assay kit. The results were calculated as the ratio of firefly luciferase activity in the miR-495-transfected cells normalized to the control cells. *P < 0.05; **P < 0.01

The *in silico* approaches TargetScan (Lewis et al., 2003) and miRanda (John et al., 2004) were used in combination to predict target genes of miR-495, and junctional adhesion molecule A (JAM-A) was identified as a potential one. The putative binding sites for miR-495 in the 3′-UTR of JAM-A mRNA are shown in Fig. 2A. The seed region (the core sequences that encompass the first 2–8 bases of the mature miRNA) of miR-495 perfectly base-pairs with 3′-UTR of JAM-A mRNA. Furthermore, the miR-495 binding sequences in the 3′-UTR of JAM-A mRNA are highly conserved across species.

To assess whether JAM-A could be regulated by miR-495, we investigated the effect of miR-495 on JAM-A protein level in MCF-7 and MDA-MB-231 cells. As shown in Fig. 2B, the level of JAM-A protein was reduced by the induction of miR-495 mimic but significantly increased by transfection with miR-495 inhibitor in both cell lines.

To ascertain whether miR-495 directly regulates JAM-A expression by binding with JAM-A 3′-UTR, the full-length 3′-UTR of JAM-A was amplified by PCR and then fused downstream of the firefly luciferase gene in a reporter plasmid. The reporter plasmid was transfected into MDA-MB-231 cells along with a transfection control plasmid (β-gal) and miR-495 mimic or inhibitor. As expected, overexpression of miR-495 resulted in approximately a 20% reduction in luciferase reporter activity, whereas inhibition of miR-495 resulted in a 1.3-fold increase in reporter activity compared with the cells transfected with control inhibitor (Fig. 2C). Furthermore, we introduced point mutations into the corresponding complementary sites in the JAM-A 3′-UTR to eliminate the predicted miR-495 binding sites. This mutated luciferase reporter was unaffected by either the overexpression or knockdown of miR-495 (Fig. 2C). In conclusion, the results demonstrate that miR-495 inhibits JAM-A expression by binding to the 3′-UTR of JAM-A.

### JAM-A expression is decreased in breast cancer tissues and is inversely correlated with the mobility of breast cancer cells

**Figure 3:**
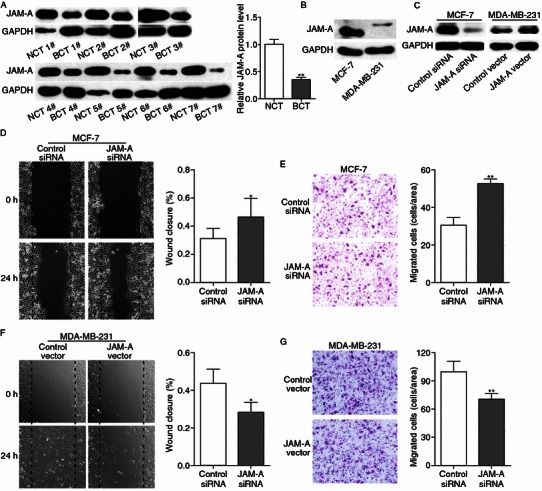
**JAM-A expression is decreased in breast cancer tissues and is inversely correlated with the mobility of breast cancer cells**. (A) Western blotting analysis and quantification of JAM-A protein levels in seven pairs of breast cancer tissue (BCT) and non-cancerous tissue (NCT) samples. (B) Western blotting analysis of JAM-A protein levels in MCF-7 and MDA-MB-231 cells. (C) Left panel: Western blotting analysis of JAM-A protein levels in MCF-7 cells transfected with control siRNA or JAM-A siRNA. Right panel: Western blotting analysis of JAM-A protein levels in MDA-MB-231 cells transfected with control plasmid or JAM-A overexpression plasmid. (D) Left panel: Representative image of wound healing assay of MCF-7 cells transfected with control siRNA or JAM-A siRNA. Right panel: Quantitative analysis of the wound closure rates. (E) Left panel: Representative image of Transwell assay of MDA-MB-231 cells transfected with control plasmid or JAM-A overexpression plasmid. Right panel: Quantitative analysis of the migration rates. (F) Left panel: Representative image of wound healing assay of MDA-MB-231 cells transfected with control plasmid or JAM-A overexpression plasmid. Right panel: Quantitative analysis of the wound closure rates. (G) Left panel: Representative image of Transwell assay of MDA-MB-231 cells transfected with control plasmid or JAM-A overexpression plasmid. Right panel: Quantitative analysis of the migration rates. *P < 0.05; **P < 0.01

MiRNAs are generally thought to have an expression pattern that is opposite to that of their targets (Olsen and Ambros, 1999). As miR-495 expression was increased in breast cancer tissue samples, we next investigated whether JAM-A protein level was decreased. After detecting the protein level of JAM-A in the same seven pairs of breast cancer and corresponding noncancerous tissue samples, we found that JAM-A protein level was dramatically lower in the breast cancer samples (Fig. 3A). Moreover, we determined the level of JAM-A protein in MCF-7 and MDA-MB-231 cells, and higher level of JAM-A protein was detected in MCF-7 cells which showed a lower level of miR-495 (Fig. 3B). These findings further suggest that the level of JAM-A protein is negatively correlated with the miR-495 level and that JAM-A expression is regulated by miR-495.

As an adhesion molecule participating in comprising tight junctions, JAM-A was reported to be associated with metastasis of breast cancer cells; however, there are conflicting reports. For example, Naik et al. (Naik et al., 2008) and Wang et al. (Wang and Lui, 2012) reported that attenuation of JAM-A contributes to breast cancer cell invasion, whereas McSherry et al. revealed that JAM-A drives breast cancer cell migration (McSherry et al., 2011). To further elucidate the function of JAM-A in regulating the mobility of breast cancer cells, loss-of-function assay was performed by transfecting an siRNA against JAM-A into MCF-7 cells. The left panel of Fig. 3C shows that the protein level of JAM-A was knocked-down in MCF-7 cells with JAM-A siRNA. Then, wound healing and Transwell assays were used to investigate MCF-7 cell migration. Within 24 h, MCF-7 cells with a lower level of JAM-A protein occupied approximately 46% of the wound, and MCF-7 cells with control siRNA covered approximately 31% of the wound, indicating that knockdown of JAM-A promoted the mobility of MCF-7 cells (Fig. 3D). Similarly, Transwell assays showed that more MCF-7 cells migrated through the porous membrane when JAM-A expression was inhibited by siRNA (Fig. 3E).

Gain-of-function assay was also conducted by transfecting JAM-A cDNA plasmid into MDA-MBA-231 cells; the increase in JAM-A protein is shown in the right panel of Fig. 3C. As expected, overexpression of JAM-A undermined the migration capability of MDA-MB-231 cells, as demonstrated by wound healing and Transwell assays (Fig. 3F and 3G). Altogether, JAM-A was found to be down-regulated in breast cancer and to negatively regulate the mobility of breast cancer cells.

### MiR-495 induces breast cancer cell migration by targeting JAM-A

**Figure 4:**
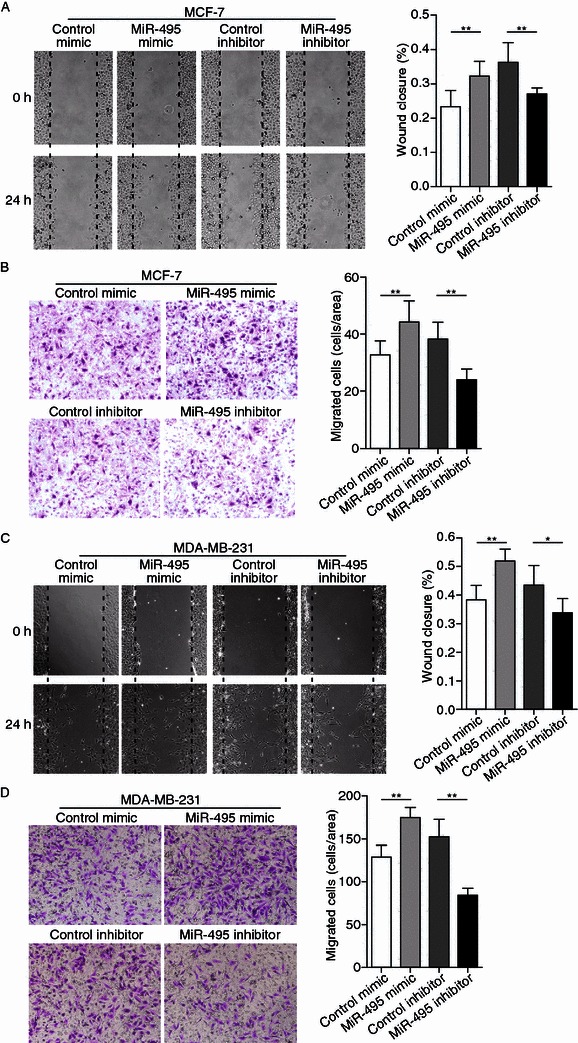
**Effects of miR-495 on breast cancer cell migration**. (A) Left panel: Representative image of wound healing assay of MCF-7 cells transfected with control mimic, miR-495 mimic, control inhibitor or miR-495 inhibitor. Right panel: Quantitative analysis of the wound closure rates. (B) Left panel: Representative image of Transwell assay of MCF-7 cells transfected with control mimic, miR-495 mimic, control inhibitor or miR-495 inhibitor. Right panel: Quantitative analysis of the migration rates. (C) Left panel: Representative image of wound healing assay of MDA-MB-231 cells transfected with control mimic, miR-495 mimic, control inhibitor or miR-495 inhibitor. Right panel: Quantitative analysis of the wound closure rates. (D) Left panel: Representative image of Transwell assay of MDA-MB-231 cells transfected with control mimic, miR-495 mimic, control inhibitor or miR-495 inhibitor. Right panel: Quantitative analysis of the migration rates. *P < 0.05; **P < 0.01

After establishing that JAM-A is involved in the migration of breast cancer cells, the biological significance of miR-495 in breast cancer was then investigated. As shown by wound healing assays in Fig. 4A, more MCF-7 cells migrated into the scratch on the cell monolayer when transfected with miR-495 mimic; consistently, knockdown of miR-495 by miRNA inhibitor showed the opposite effects. The same biological function of miR-495 was found in MDA-MB-231 cells (Fig. 4C). In addition, Transwell assays revealed a significant increase in cells migrating through the membrane when MCF-7 cells and MDA-MB-231 cells were transfected with miR-495 mimic (Fig. 4B and 4D). As anticipated, the mobility of MCF-7 and MDA-MB-231 cells was clearly inhibited by transfection with miR-495 inhibitor, shown by Fig. 4B and 4D, respectively.

**Figure 5:**
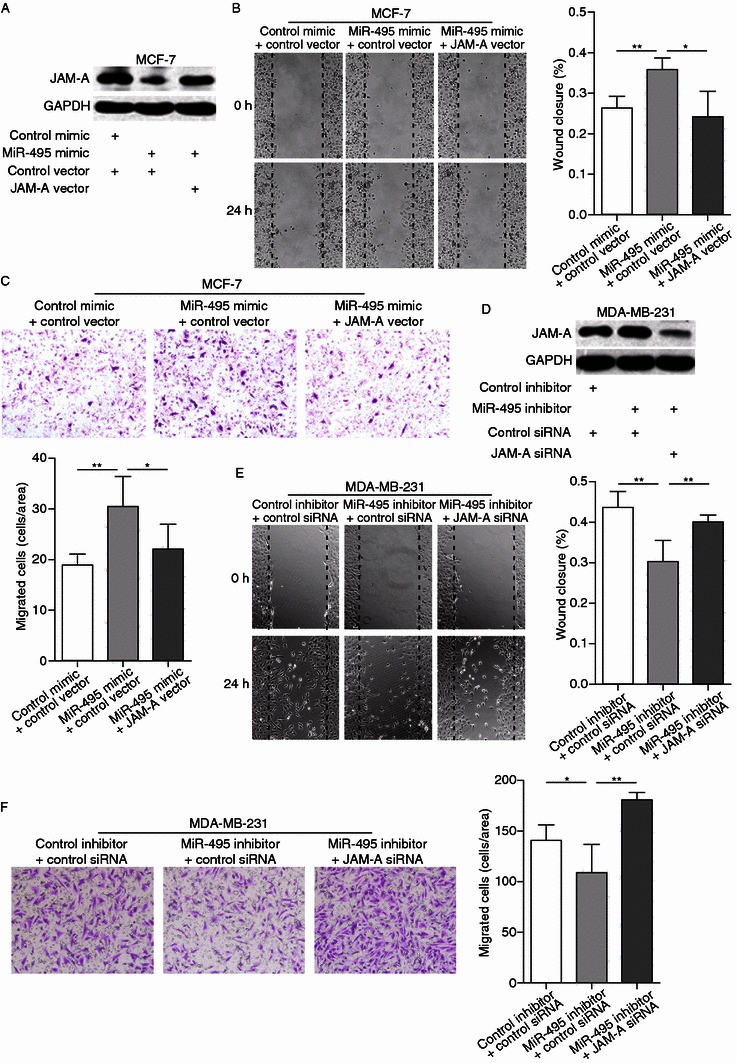
**MiR-495 induces breast cancer cell migration by targeting JAM-A**. (A) Western blotting analysis of the protein levels of JAM-A in MCF-7 cells transfected with control mimic plus control plasmid, miR-495 mimic plus control plasmid or miR-495 mimic plus JAM-A overexpression plasmid. (B) Left panel: Representative image of wound healing assay of MCF-7 cells transfected with control mimic plus control plasmid, miR-495 mimic plus control plasmid or miR-495 mimic plus JAM-A overexpression plasmid. Right panel: Quantitative analysis of the wound closure rates. (C) Top panel: Representative image of Transwell assay of MCF-7 cells transfected with control mimic plus control plasmid, miR-495 mimic plus control plasmid or miR-495 mimic plus JAM-A overexpression plasmid. Bottom panel: Quantitative analysis of the migration rates. (D) Western blotting analysis of the protein levels of JAM-A in MDA-MB-231 cells transfected with control inhibitor plus control siRNA, miR-495 inhibitor plus control siRNA or miR-495 inhibitor plus JAM-A siRNA. (E) Left panel: Representative image of wound healing assay of MDA-MB-231 cells transfected with control inhibitor plus control siRNA, miR-495 inhibitor plus control siRNA or miR-495 inhibitor plus JAM-A siRNA. Right panel: Quantitative analysis of the wound closure rates. (F) Left panel: Representative image of Transwell assay of MDA-MB-231 cells transfected with control inhibitor plus control siRNA, miR-495 inhibitor plus control siRNA or miR-495 inhibitor plus JAM-A siRNA. Right panel: Quantitative analysis of the migration rates. *P < 0.05; **P < 0.01

To further confirm that the effects of miR-495 are mediated by repression of JAM-A in breast cancer cells, knockdown of JAM-A in MCF-7 cells by miR-495 mimic was restored by transfecting JAM-A cDNA vector, the expression of which is not regulated by miR-495 due to its lack of the 3′-UTR (Fig. 5A). Compared with the cells transfected with miR-495 mimic and control vector, MCF-7 cells transfected with miR-495 mimic and JAM-A vector exhibited impaired migration ability (Fig. 5B and 5C). Moreover, JAM-A siRNA was transfected into MDA-MB-231 cells to counteract the increase of JAM-A protein caused by miR-495 inhibitor (Fig. 5D). Fig. 5E and 5F show that the inhibition of MDA-MB-231 cell migration by miR-495 inhibitor can be reversed by JAM-A siRNA. Taken together, these results demonstrated that miR-495 promotes breast cancer cell migration by inhibiting JAM-A.

## Discussion

MiRNAs are a class of small, non-coding RNAs that regulate the expression of specific mRNAs by either translational inhibition or mRNA degradation (Bartel, 2004; Fabian et al., 2010). More than 50% of miRNAs are reported to be located in cancer-associated genomic break points (Calin et al., 2004), and these miRNAs can function as tumor suppressor genes or oncogenes, depending on the target genes regulated by the miRNA (Croce, 2009). MiR-495 was reported to act as an oncogene in multiple models of cancer. For example, up-regulation of miR-495 contributes to lower MAT1 expression and enhanced tumorigenesis in hepatocellular carcinoma (HCC) and may represent a potential target for HCC therapy (Yang et al., 2013). In addition, up-regulation of miR-495 by E12/E47 in breast cancer stem cells promotes oncogenesis and hypoxia resistance via the down-regulation of E-cadherin and REDD1 (Hwang-Verslues et al., 2011). However, miR-495 was also shown to suppress tumorigenesis in different types of cancer. For example, miR-495 functions as a tumor suppressor by targeting essential leukemia-related genes in MLL-rearranged leukemia (Jiang et al., 2012) and by down-regulating cyclin-dependent kinase 6 in glioblastoma multiforme cells (Chen et al., 2013). Moreover, miR-495 inhibits the migration of gastric cancer cells and lung cancer cells by targeting PRL-3 and MTA 3, respectively (Chu et al., 2014; Li et al., 2012). Although the function of miR-495 is still controversial in different types of cancer, in this study, we found that the expression of miR-495 was up-regulated in clinical breast cancer samples, indicating that miR-495 may be associated with the progression of breast cancer.

Breast cancer cell line MCF-7 was classified into luminal A subtype, which exhibited a characteristic epithelial cobblestone-like morphology with high expression of cell-cell adhesion molecules such as E-cadherin, and has been identified as a representative breast cancer cell line with low-invasive ability. By contrast, MDA-MB-231 was classified into claudin-low subtype, and this cell line displayed a high invasive potential with the characteristics of epithelial-mesenchymal transition (EMT), such as expressing a high level of vimentin (Holliday and Speirs, 2011; Neve et al., 2006). In the present study, the highly tumorigenic breast cancer cell line MDA-MB-231 was found to present a high level of miR-495 when being compared with the less tumorigenic cell MCF-7. These results suggest that miR-495 is positively associated with the malignant phenotypes of breast cancer, including the migration of breast cancer cells, and may serve as a marker of potential progression of the cancer.

According to the bioinformatics analysis, junction adhesion molecule A (JAM-A, JAM-1, F11 receptor, CD321) was predicted as a potential target gene of miR-495. Consistently, the level of JAM-A protein was decreased in human breast cancer tissues, which is coincident with the up-regulation of miR-495. Furthermore, MCF-7 cells with a lower level of miR-495 expressed a higher level of JAM-A protein than MDA-MB-231 cells. Moreover, Western blotting analysis showed that miR-495 inhibits JAM-A translation in breast cancer cells. We also found that luciferase activity of the reporter plasmid containing JAM-A 3′-UTR can be remarkably decreased by miR-495 mimic, while knockdown of miR-495 in MDA-MB-231 cells resulted in an increase of luciferase activity. These results proved that miR-495 can fine-tune the expression of JAM-A through binding to 3′-UTR of JAM-A.

Originally characterized by the monoclonal antibody F11 on platelets, JAM-A is a type I transmembrane glycoprotein involved in numerous biological and pathological processes (Mandell and Parkos, 2005). JAM-A is a cell-adhesion protein predominantly expressed at the tight junctions of epithelial cells, including those of the mammary epithelium (Feigin and Muthuswamy, 2009). Under normal circumstances, JAM-A participates in the formation of tight junctions (Schneeberger and Lynch, 2004). The disruption of focal adhesion is associated with EMT, which leads to the promotion of cell migration (Lee et al., 2006). However, the role of JAM-A in the migration of tumor cells remains a controversial issue. Koshiba et al. reported that JAM-A expression was reduced in high-grade or advanced endometrial carcinoma and may constitute a poor prognostic factor (Koshiba et al., 2009). In addition, Fong et al. found that low JAM-A expression is associated with metastasis and poor survival in pancreatic cancer (Fong et al., 2012). Nevertheless, another report published recently showed that high JAM-A expression in non-small cell lung cancer (NSCLC) tissues is positively correlated with NSCLC progression (Zhang et al., 2013). These data suggest that JAM-A expression is regulated in a tissue-dependent manner. Additionally, conflicting data about the function of JAM-A in regulating cancer cell migration have been reported with regard to breast cancer. Naik et al. initially reported that up-regulation of JAM-A expression decreased migration and invasion in breast cancer cells, whereas JAM-A knockdown enhanced invasiveness (Naik et al., 2008). It was therefore hypothesized that the loss of JAM-A may correlate with poor clinical prognosis. Similar results were reported by Wang et al., who demonstrated that transforming growth factor-β1 (TGF-β1) induced breast cancer cell invasion by down-regulating JAM-A expression (Wang and Lui, 2012). However, McSherry and colleagues reported that the expression of JAM-A is positively correlated with poor prognosis in invasive breast cancer patients, indicating that JAM-A promotes cell motility in breast cancer by activating Rap 1 GTPase (McSherry et al., 2011). In our study, loss-of-function assay using MCF-7 cells transfected with JAM-A siRNA showed that knockdown of JAM-A promotes the migration of breast cancer cells, whereas gain-of-function assay by overexpressing JAM-A in MDA-MB-231 cells showed that JAM-A overexpression attenuates migration. These results suggest the role of JAM-A as a negative regulator of migration in breast cancer cells.

Regarding the mechanisms of the down-regulation of JAM-A protein in breast cancer, previous report showed that TGF-β1 inhibits JAM-A gene transcription via the activation of Smads (Wang and Lui, 2012). Considering that the 3′-UTR of human JAM-A is as long as 3.6 kb, we hypothesized that JAM-A expression may be down-regulated by miRNAs, such as miR-495, in breast cancer; indeed, a negative correlation between the expression of miR-495 and JAM-A was established in the present study. The overexpression of miR-495 promoted the mobility of breast cancer cells by down-regulating JAM-A. Additionally, the restoration of JAM-A protein by expressing JAM-A cDNA vector, which lacked 3′-UTR and thus not regulated by miRNAs, reversed the migration promotion exerted by miR-495, further confirming that miR-495 regulates the migration of breast cancer cells by targeting JAM-A. Moreover, comparison of the mobility of MDA-MB-231 cells transfected with miR-495 inhibitor plus control siRNA or miR-495 inhibitor plus JAM-A siRNA revealed that miR-495 inhibitor down-regulated the migration of breast cancer cells by regulating JAM-A expression.

Taken together, our data reveal a new role for miR-495 as an oncogenic miRNA in breast carcinogenesis. Identification of the miR-495-targeting JAM-A pathway provides a potential new therapeutic target in the treatment of breast cancer.

## Materials and methods

### Ethics statement

Breast cancer tissue and paired adjacent noncancerous tissue samples were collected from the Affiliated Drum Tower Hospital of Nanjing University Medical School and the Affiliated Jinling Hospital of Southern Medical University (Nanjing, China). Written informed consent was obtained from all patients. The Ethics Committee of Nanjing University approved all aspects of this study. The tissue fragments were immediately frozen in liquid nitrogen at the time of surgery and stored at −80°C. The clinical features of the patients are listed in Table S1.

### RNA isolation and quantitative real time-PCR (qRT-PCR)

Total RNA was extracted from cultured cells or tissue samples with TRIzol Reagent (Invitrogen) according to the manufacturer’s instructions. Assays for miR-495 quantification were conducted by using gene-specific TaqMan miRNA Assay Probes (Applied Biosystems, Foster City, CA). After the real-time PCR reaction, the cycle threshold (C_T_) data were determined using fixed threshold settings; the mean C_T_ was determined from triplicate PCRs. A comparative C_T_ method was used to compare each condition to the controls. The U6 snRNA was used as an internal control, and the relative amount of miR-495 normalized to U6 was calculated using the equation 2^−ΔΔCT^, where ΔΔC_T_ = (C_T miR-495_ − C_T U6_)_target_ − (C_T miR-495_ − C_T U6_)_control_.

### Overexpression or knockdown of JAM-A

A full-length *JAM-A* cDNA expression plasmid (EX-U0777-M02) lacking the 3′-UTR was purchased from GeneCopoeia (Rockville, MD, USA), and the empty plasmid served as the negative control. The siRNA sequence targeting human *JAM-A* mRNA was designed and synthesized by RiboBio (Guangzhou, China), with a scrambled siRNA included as negative control. The sequences of the *JAM-A* siRNA were as follows: 5′-GGAUAGUGAUGCCUACGAAdTdT-3′ (sense); 5′-dTdTCCUAUCACUACGGAUGCUU-3′ (antisense).

The *JAM-A* overexpression plasmid or siRNA was transfected into MCF-7 or MDA-MB-231 cells using Lipofectamine 2000 (Invitrogen) according to the manufacturer’s instructions. The concentration for transfection of *JAM-A* cDNA plasmid or siRNA was 0.05 µg/mL or 50 nmol/L, respectively.

### Overexpression or knockdown of miR-495

The overexpression or knockdown of miR-495 was accomplished by transfecting cells with a synthetic miR-495 mimic or inhibitor purchased from Genephama (Shanghai, China). The transfection concentrations were 50 nmol/L for the miR-495 mimic and 200 nmol/L for the miR-495 inhibitor, which were also adopted when the control mimic or inhibitor was transfected.

### Western blotting

Western blotting was carried out as described previously (Cao et al., 2014). An anti-JAM-A antibody was obtained from Epitomics (#1840-1, Burlingame, CA, USA). An antibody against GAPDH, as the control, was obtained from Santa Cruz Biotechnology (Santa Cruz, CA, USA).

### Luciferase reporter assay

The entire 3′-UTR of human JAM-A was amplified by PCR using human genomic DNA as the template, and the PCR products were inserted into the p-MIR-reporter plasmid (Ambion, Austin, TX, USA). The insertion was confirmed by sequencing. To test the binding specificity, the sequences that interact with the miR-495 seed sequence were mutated (from GUUUGUU to CAAACAA), and the mutant JAM-A 3′-UTR was inserted into an equivalent luciferase reporter plasmid. For the luciferase reporter assay, MDA-MB-231 cells were seeded in 24-well plates, and each well was transfected with 0.2 µg of luciferase reporter plasmid, 0.4 µg of β-galactosidase (β-gal) expression plasmid and 40 pmol of miR-495 mimic, 120 pmol of miR-495 inhibitor or scrambled negative control RNAs using Lipofectamine 2000 (Invitrogen). The β-gal plasmid was used as the transfection control. At 24 h after transfection, the cells were harvested and analyzed for luciferase activity using luciferase assay kits (Promega, Madison, WI, USA).

### Wound healing assays

Cell migration was assessed in classical wound healing assays with some modifications (Rodriguez et al., 2005). Briefly, cells were seeded in 6-well plates and transfected when they were attached. After transfection, the cells were allowed to culture to confluence. Then, the cell layer was gently wounded using a plastic pipette tip (P200) and rinsed with PBS before the culture medium was replaced. The bottom of the wells was marked to indicate where the initial images of the wounded area were captured. At 24 h of incubation, images (10×) of the same areas were recorded using a photomicroscope (BX51 Olympus, Japan), and closure of the wounds was processed using Image-Pro Plus 6.0.

### Transwell assays

The migration ability of cells was also tested in a Transwell Boyden Chamber (6.5 mm, Costar, USA). The polycarbonate membranes (8-μm pore size) on the bottom of the upper compartment of the Transwells were coated with 1% human fibronectin (R&D Systems 1918-FN, USA). At 24 h after transfection, the cells were harvested, counted and suspended in FBS-free DMEM medium. Then, 3 × 10^4^ cells in 200 µL DMEM medium were added to each upper chamber; 0.6 mL of DMEM with 10% FBS was added to the lower compartment, and the Transwell-containing plates were incubated at 37°C and 5% CO_2_ for 8 h. After incubation, the cells that had entered the lower surface of the membrane were fixed with 4% paraformaldehyde for 20 min at room temperature, washed 3 times with distilled water and stained with 0.1% crystal violet in 0.1 mol/L borate and 2% ethanol for 15 min at room temperature. The non-migrant cells remaining on the upper surface of the filter membrane were scraped off gently with a cotton swab. The lower surfaces (with migrant cells) were captured using a photomicroscope (5 fields per chamber) (BX51 Olympus, Japan), and the cells were counted blindly.

### Statistical analysis

All images of Western blotting, wound healing assays and Transwell assays are representative of at least three independent experiments. Quantitative RT-PCR and the luciferase reporter assay were performed in triplicate, and each experiment was repeated at least three times. The results are presented as the mean ± SD. The differences between groups were calculated using Student’s *t*-test, and *P* < 0.05 was defined as statistically significant.

## Electronic supplementary material

Below is the link to the electronic supplementary material.

Supplementary material 1 (PDF 23 kb)

## Abbreviations

EMT: epithelial-mesenchymal transition
HCC: hepatocellular carcinoma
JAM-A: junctional adhesion molecule A
miRNAs: microRNAs
qRT-PCR: quantitative real time-PCR
TGF-β1: transforming growth factor-β1
