# Supplementary material for: MicroRNA-495 induces breast cancer cell migration by targeting JAM-A
Source: Protein Cell. 2014 Jul 30;5(11):862–72. doi: 10.1007/s13238-014-0088-2 (PMC4225486; doi:10.1007/s13238-014-0088-2)
Supplement: Supplementary file 1 — Supplementary material 1 (PDF 23 kb) [file 13238_2014_88_MOESM1_ESM.pdf]

Supplementary Table 1. Clinical features of breast cancer patients.

Patients' characteristics						
Case No.	Clinical History	Gender	Age (years)	TNM Stage	HER-2 Status	ER
BC #1	IDC	Female	48	II	2+	3+
BC #2	IDC	Female	44	II	2+	2+
BC #3	ILC	Female	58	II	2+	3+
BC #4	IDC	Female	71	I-II	-	-
BC #5	IDC	Female	30	II	3+	-
BC #6	IDC	Female	51	II	-	-
BC #7	IDC	Female	49	IV	-	3+
